# Potential of In Vitro Culture of *Scutellaria baicalensis* in the Formation of Genetic Variation Confirmed by ScoT Markers

**DOI:** 10.3390/genes13112114

**Published:** 2022-11-14

**Authors:** Jacek Gawroński, Magdalena Dyduch-Siemińska

**Affiliations:** Department of Genetics and Horticultural Plant Breeding, Institute of Plant Genetics, Breeding and Biotechnology, University of Life Sciences in Lublin, Akademicka 15 Street, 20-950 Lublin, Poland

**Keywords:** indirect organogenesis, genetic diversity, medicinal plant, molecular marker, plant tissue culture, somaclonal variation

## Abstract

The in vitro culture technique can be used for micropropagation of medicinal plants as well as for creating genotypes with an improved profile of phytochemical compounds. For this purpose, somaclonal variability may be used for the induction of genetic diversity among regenerants. The paper presents a protocol for obtaining *Scutellaria baicalensis* regenerants by indirect organogenesis and the assessment of their genetic variability with the use of start codon-targeted markers. The most intense process of indirect shoot organogenesis was observed on Murashige and Skoog medium supplemented with kinetin and 6-Benzylaminopurine (0.5 mg × dm^−3^ each)—7.4 shoot per explant on average. The callogenesis process occurred on the medium supplemented with TDZ, while the medium supplemented with GA_3_ allowed for direct shoot organogenesis and was used for the micropropagation of regenerants. In the analysis of plantlets obtained by indirect organogenesis, 11 ScoT markers generated a total of 130 amplicons, 45 of which were polymorphic. This analysis showed genetic diversity of regenerants in relation to the donor plant as well as within them, with mean similarity among the analyzed genotypes at the level of 0.90. This study confirms that the use of in vitro cultures allows for the possibility to generate genetic variability in *Scutellaria baicalensis*, which can be effectively revealed with the use of the SCoT marker.

## 1. Introduction

*Scutellaria baicalensis* Georgi. (common name—Baikal skullcap) is a widespread medicinal plant of the family *Lamiaceae*. It belongs to the genus *Scutellaria*, subfamily *Scutellarioideae*. The species is diploid (2n = 18) and belongs to entomophilous cross-pollinated plants [1,2]. Baikal skullcap is one of the 50 essential herbs of traditional Chinese medicine that have been used since ancient times to treat inflammation, high blood pressure, bacterial and viral infections, and to improve the overall health of the body. *S. baicalensis* contains many active substances, the main source of which is the root and, to a lesser extent, the aerial parts of the plant [3]. Baicalin, baicalein, vogonoside and vogonin are the most popular flavonoids, and their content in *S. baicalensis* root is as high as 20%, of which approximately 12–17% constitute baicalin and 3–4% constitute vogonoside [4]. In addition to flavonoids, *S. baicalensis* contains volatile oils, which are mainly responsible for the scent and sweet taste, and additionally exhibit antibacterial activity against Gram-positive and Gram-negative bacteria [5]. *S. baicalensis* also contains over 100 diterpenoids, mainly in the aerial part, which exhibit antibacterial and antiviral activity [6]. An important aspect in the cultivation of Baikal skullcap is the appropriate selection of conditions that have a significant impact on the content of active ingredients in the plant. In vitro cultures are an alternative to conventional methods of vegetative reproduction. Culturing plant material in vitro can induce or reveal variation between cells, tissues and organs, thereby creating variability within cultures or between regenerated plants (somaclonal variation). Some or all of the somaclons may differ phenotypically and genetically from the donor plants from which the culture was derived. Variability of this type, which usually occurs spontaneously and is largely uncontrolled, may be the result of genetic and epigenetic changes occurring in cells in vivo as well as during in vitro culture [7]. In vitro culture alone or in combination with mutagenesis induced by physicochemical and biological factors can be used to produce plants with increased genetic variability and mutants as a potential source of new commercial varieties. For this reason, plants regenerated from tissue, organ, callus, or protoplast cultures and through somatic embryogenesis may show variability at the phenotypic and genotypic level [8]. This type of variability can be studied using molecular markers. In 2009, Collard and Mackill [9] developed a new alternative and repeatable technique based on start codon-targeted (SCoT) markers. These markers are based on DNA and target the conserved ATG translation start codons. The SCoT system has been successfully used to assess genetic diversity or identify varieties and map quantitative trait loci in different species, generating high polymorphism and repeatability of the results.

The main aim of the study was: (i) to obtain regenerants of *S. baicalensis* through indirect organogenesis; and (ii) the assessment of genetic variation in plants regenerated in vitro using SCoT markers. The study also optimized the procedure for obtaining *S. baicalensis* regenerants and their multiplication in vitro, which will enable access to ready protocols for efficient propagation of the analyzed species.

## 2. Materials and Methods

In order to assess the possibility of inducing genetic variation during in vitro culture of the tested species, a two-step procedure for obtaining regenerants was applied.

### 2.1. Procedure for Obtaining Regenerants

#### 2.1.1. Callus Induction and Shoot Regeneration

**Figure 1:**
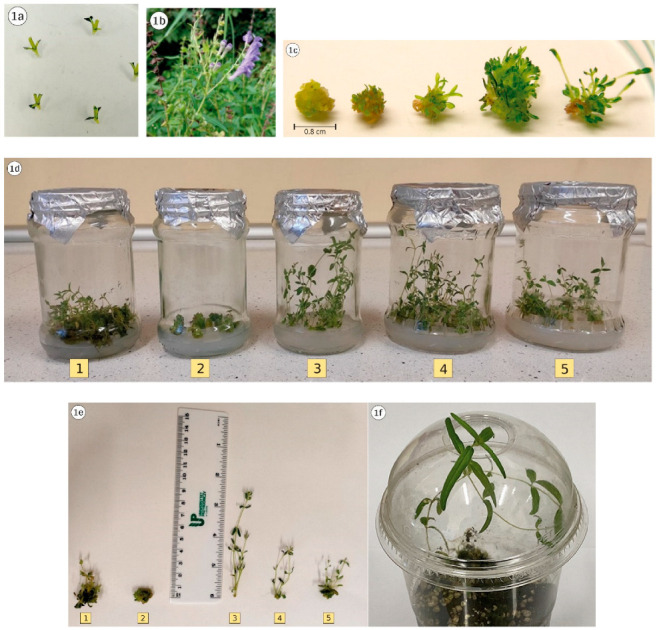
In vitro regeneration of *Scutellaria baicalensis* on Murashige and Skoog (MS) media. (**1a**)—primary explants used to establish the culture; (**1b**)—Donor plants in in vivo condition; (**1c**)—Indirect organogenesis from nodal explants MS medium supplemented with KIN + BAP (0.5 mg × dm^-3^ each); (**1d**)—Cultures on the media according to Table 1 after 42 days. (**1e**)—Plantlet on the media according to Table 1 after 42 days. (**1f**)—Plantlet acclimatization in pot containing soil and perlite in a 2:1 ratio.

**Figure 2:**
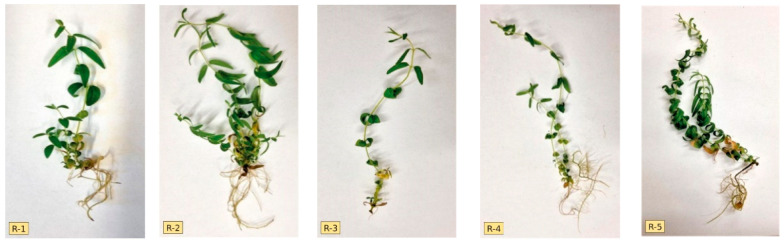

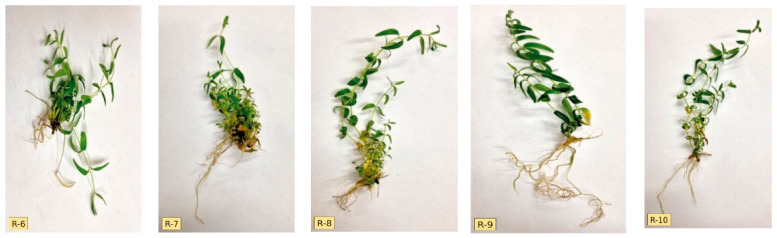
Phenotypic diversity of regenerants (from R-1 to R-10) on medium number 3 after 6 weeks of the culture.

At this stage, through the use of nutrient supplementation with different types and concentrations of plant growth regulators (PGRs), it was determined which ones had the greatest effect on the process of indirect shoot organogenesis, i.e., callus tissue formation and shoot regeneration. The regenerated shoots could potentially show variation at the DNA level due to the genetic instability of the callus tissue. These shoots were obtained in such a way that the mature *S. baicalensis* plants grown at the Experimental Farm of the Department of Vegetable and Medicinal Plants of the University of Life Sciences in Lublin (51°14′53″ N, 2°34′13″ E) were used as a source material for the experiment, i.e., donor plants (DP—Figure 2). Directly after harvest, the explants were transported to the in vitro culture laboratory at the Institute of Plant Genetics, Breeding and Biotechnology, University of Life Sciences in Lublin. Healthy shoots with 10–12 nodes were harvested from field-grown plants and washed under tap water for an hour. Shoots were subsequently rinsed for 15 min in distilled water with a drop of Tween-20 on a stirrer. Shoots were sterilized with 75% ethanol for 1 min and 1% sodium hypochlorite for 5 min. Shoots were then washed three times (5 min each) in sterile distilled water and cut into fragments to obtain nodal explants (Figure 1). Explants were transferred to jars containing approximately 15 mL Murashige and Skoog (MS) [10] medium with different types and concentrations of plant growth regulators (PGRs). The type and concentration of PGs used are listed in Table 1. The medium composition was as follows: Murashige and Skoog basal medium supplemented with sucrose (30.0 g dm^−3^), thiamine (0.4 g × dm^−3^), pyridoxine (0.5 mg × dm^−3^), nicotinic acid (0.5 mg × dm^−3^), inositol (100 dm^−3^), PGRs and agar-agar (8.0 g × dm^−3^). (Sigma-Aldrich—St. Louis, MO, USA). For the experiment, the pH of the medium was adjusted to 5.8 with 1 M NaOH and 1 M HCl before autoclaving at 121 °C for 20 min. After initiation, the explants were cultured under 40 μmol m^−2^ s^−1^ light provided by cool white fluorescent tubes with a 16-h photoperiod and a temperature of 21 °C ± 2 °C. The experiment was carried out in triplicate. One replicate consisted of three jars of five explants each. Observations of the number of propagated shoots, their average length, average number of nodes and callus formation were carried out after 42 days of the culture.

#### 2.1.2. Shoot Multiplication of Regenerants

The hypothesis concerning the genetic diversity of regenerants obtained in the first stage was verified in the second stage by conducting direct shoot organogenesis, and the obtained plants were intended for DNA analysis. To this end, 10 randomly selected shoots regenerated on MS medium supplemented with 0.5 mg × dm^−3^ BAP (6-Benzylaminopurine) and kinetin 0.5 mg × dm^−3^ were cut into nodal fragments and used as secondary explants. Three nodal explants were collected from the selected regenerant for individual multiplication. Explants were placed on an MS medium supplemented with GA_3_. The number of propagated shoots, their average length, the average number of nodes and root system development stage were assessed after 6 weeks of the culture. After this time, the plants were ready for the process of acclimatization.

### 2.2. Acclimatization

In vitro-rooted plantlets were removed from the jar. Their roots were washed with running tap water to remove the medium. Regenerants were inserted into plastic vessels filled with soil and perlite in a 2:1 ratio and kept in a growth chamber at 23–25 °C and 16-h photoperiod for 20 days. During this time, the high relative humidity in the vessel was gradually reduced by removing the cover. The plants were subsequently transferred to a greenhouse for further acclimatization.

### 2.3. Molecular Assays

#### 2.3.1. DNA Extraction

Genomic DNA was isolated from a fragment of a shoot with leaves of the mother plant and regenerated plants after the multiplication process was completed (Section 2.2). DNA was extracted following the CTAB method described by Doyle and Doyle [11]. The DNA concentration was determined using a Nanodrop spectrophotometer (Thermo Scientific). All test samples were diluted to a final concentration of 25 ng μL^−1^.

#### 2.3.2. SCoT Analysis

To perform the genetic analysis of *S. baicalensis*, the PCR was optimized for the SCoT markers tested. The optimization involved determining the optimal magnesium ion concentration, which affects polymerase performance. Thus, primers that initiated stable amplification of clearly separated bands could be selected. Eleven 18-base primers selected from 20 arbitrary primers were used for PCR amplification. DNA amplification of SCoT markers was carried out in a final volume of 10 μL containing 0.5 U of Taq DNA Polymerase (Fermentas), 0.8 μL of oligonucleotide primer (0.8 μM), 1 μM dNTPs, 1 × PCR buffer with 1.5 mM MgCl_2_, and 25 ng of genomic DNA as a template. Amplification was performed in a gradient thermal cycler (Biometra GmbH) with the following reaction conditions: initial predenaturation at 94 °C for 3 min, followed by 35 denaturation cycles at 94 °C for 1 min, annealing at 50 °C for 1 min, and extension at 72 °C for 2 min. The final extension was carried out for 5 min at 72 °C with the holding temperature of 4 °C. To verify reproducibility, the primers were tested twice on the same sample.

PCR products were electrophoresced in 1.5% agarose gels stained with ethidium bromide at constant voltage (3 V cm^−1^) until bromophenol blue/loading dye migrated to the other end of the gel. The gel was visualized in a UV transilluminator and photographed using a GeneSnap ver. 7.09 (SynGene) gel documentation system. NZYDNA Ladder III (NZYTech) was used to establish the molecular weight of the products. Among obtained SCoT products, only reproducible and clear fragments were scored from the photographs. Bands detected in analyzed genotypes and scored as present (1) or absent (0) were considered polymorphic profiles, while specific bands were restricted to a specific individual. Indistinct or weak bands were excluded from the analysis.

### 2.4. Statistical Analysis

Statistical analysis of the results of shoot multiplication of regenerants was carried out using ANOVA, and the significance of differences between mean values was calculated using Duncan’s multiple range tests performed at *p* < 0.05. The similarity coefficient between the studied genotypes in the SCoT analysis was assessed according to the Dice formula [12]. A cluster analysis was conducted using the UPGMA (unweighted pair-group method with arithmetic mean) distance method implemented in the PAST software [13].

## 3. Results and Discussion

### 3.1. Callus Induction and Shoot Regeneration

Phytohormones applied at the regeneration stage caused different directions of culture development (Table 1, Figure 1).

The process of direct shoot organogenesis was observed—medium 3, indirect shoot organogenesis (intensive callus formation with intensive shoot production, as well as the formation of a small amount of callus with simultaneous shoot formation)—medium 1 and 5, moreover formation of only callus tissue—medium 2. Since the prerequisite for obtaining genetic differentiation among regenerants is their formation via callus tissue, it should be pointed out that its formation was most intense on the medium containing KIN + BAP (0.5 mg × dm^−3^ each) and the medium with TDZ (0.5 mg × dm^−3^) addition. In contrast, small amounts of callus were observed on media with a combination of GA_3_ + 2iP (0.5 mg × dm^−3^ each) and with 2iP only (0.5 mg × dm^−3^). With respect to the medium containing TDZ (0.5 mg × dm^−3^), intensive formation of callus tissue was observed, on which subsequently a large number of shoot buds formed; unfortunately, they were not capable of regenerating shoots. Despite the statement of Guo et al. [14] that TDZ induced shoot regeneration in many plant species of the genus *Scutellaria*, (Table 1), such an effect was not recorded. Similarly, in a study of Ozdemir et al. [15], TDZ effectively stimulated the formation of callus tissue on various types of explants derived from seedlings of this species. However, on some of them, although shoot buds were clearly visible on the explants, they did not show any shoot regeneration. In a study by Stojakowska et al. [16], nodal explants on TDZ-containing media produced caulogeniccalli, and those obtained at a concentration of 0.5 µM TDZ developed numerous shoot buds, but only a few were capable of regenerating shoots. In contrast, Zhang et al. [17] reported that calli induced by 0.3 mg × dm^−3^ TDZ produced shoots directly, while those obtained with a higher TDZ concentration required a change of medium composition to initiate the process. In turn, Gharari et al. [18] indicated that by increasing TDZ concentrations (0.5 mg × dm^−3^), the response rate of explants for shoot induction increased. Therefore, in connection with the aim of the research, it is necessary to indicate that potential genetic variability should be sought among the regenerants obtained on medium containing the combination of KIN + BAP (0.5 mg × dm^−3^ each). Nevertheless, it should be noted that, as shown by other authors, the process of indirect shoot organogenesis can be carried out using various combinations of phytohormones. According to Gharari et al. [19], stem explants of *S. araxensis* allowed efficient shoot organogenesis through the callus stage, and obtaining up to 18 new shoots per explant on medium containing 0.5 mg × dm^−3^ BAP in addition to 0.5 mg × dm^−3^ IBA. Hwang et al. [20] reported that callus induction in *S. baicalensis* occurred in the presence of 1-Naphthaleneacetic acid (NAA) 1 mg × dm^−3^ plus BAP mg × dm^−3^, while Trivedi et al. [21] showed that relatively high concentrations of TDZ (4 mg × dm^−3^) stimulated this process, and subsequently NAA 1 mg × dm^−3^ was sufficient for shoot regeneration. In contrast, according to a study by Ozdemir et al. [22], in *S. orientalis* subsp. *bicolor*, there was the possibility of callus regeneration in the presence of BAP alone or in combination with NAA.

Considering the number of shoots produced by the explant, the medium containing KIN + BAP (0.5 mg × dm^−3^ each) proved to be most optimal, and an average of 7.4 new shoots were obtained on this substrate. Slightly lower values, although statistically not significantly different from the above, were recorded on medium enriched with 2iP (0.5 mg × dm^−3^)—6.0 shoots per explant, and the medium with GA_3_ and 2iP (0.5 mg × dm^−3^ each)—4.8 shoots per explant. In contrast, only 2.1 shoots per explant were obtained on substrate with GA_3_. The latter is generally known for its effect in the direction of shoot elongation [19], and only caused the development of two axillary buds present on the explant, with the length of the resulting shoots being greater compared to those obtained on the other media. Stem elongation in the presence of GA_3_ also resulted in the development of an average of one internode more on the shoot compared to the other PGRs applied. Nevertheless, when using the regenerant cultures obtained at this stage for further multiplication using nodal explants, the highest culture efficiency can be obtained using KIN + BAP medium (7.4 × 3.0 = 22.2 nodal segments) and 2iP (6.0 × 3.5 = 21 nodal segments). This stage of culture could be summarized by the statement that the PGRs used in the medium induced a differential response of the explants (same genotype), resulting in the possibility of genetic variability occurrence among regenerants, particularly those obtained on medium 1.

### 3.2. Shoot Multiplication of Regenerants

Due to the fact that the process of direct regeneration from axillary buds was observed on the medium with GA_3_ addition, it was applied in the second stage of culture, which required limiting the possibility of variability, and as reported by Ngezahayo and Liu [23], axillary bud proliferation was the most frequently used, and also considered the most suitable to guarantee the genetic stability of the regenerated plants. Since a genetically diverse group of genotypes is likely to be evaluated at this stage, it should be assumed that they would show a slightly different morphogenetic response to the applied medium (the same for all genotypes). The data in Table 2 and Figure 2 clearly show the differentiation of the regenerants at the phenotypic level expressed by the coefficient of variation, whose value was the highest for the mean shoot number, while slightly lower for the other two traits—mean shoot length and mean number of nodes per shoot. This especially reflected the variability in the number of shoots, which ranged from 2.0 to 9.4. Variability regarding this trait was also observed in other species of the genus. The average number of shoots in *S. alpina* and *S. altissima* was 5.4 and 2.0, respectively, and was obtained on a medium with identical PGR composition [24]. The observed differences could be due to the fact that the studied species reacted differently to the applied media. On the other hand, the identical number of shoots (an average of 4.5) obtained on MS medium supplemented with meta-Topolin + NAA for both *S. barbata* and *S. racemosa*, in this case indicated a similar response of each species [25]. The interactions between genotype, a type and PGR concentration in the assessment of morphogenetic capacity, were described previously by Dyduch-Siemińska [26]. Comparing the mean value of shoot length and mean number of nodes per shoot of the analyzed regenerants in relation to the donor plant cultured on the same medium (Table 1—medium 3), genotypes R5 and R9 could be considered the most distinct characterized by advantageous morphogenetic potential, while R7 was particularly unfavorable. Regenerants after this stage of culture were fully prepared for the acclimatization process and after its completion (Figure 1f), they were planted at the Experimental Farm of the Department of Vegetable and Medicinal Plants to evaluate their phytochemical properties.

### 3.3. Molecular Assays

Since DNA methylation, amplification, activation of transposable elements, polyploidy, changes in chromosome number or DNA sequence can be affected by in vitro culture conditions and consequently lead to genetic changes in regenerated plants [27,28], it is necessary to monitor the occurrence of somaclonal variations during in vitro propagation and assess the genetic constitution and variability of the plants regenerated in vitro. The genetic diversity was successfully detected in the genus *Scutellaria* and mainly concerned the assessment of the genetic diversity of the study’s wild and cultivated populations using RAPD markers [29,30,31], ISSR markers [32,33], and SSR (simple sequence repeat) markers [34]. The use of the aforementioned markers to assess the variability of Baikal skullcap genotypes obtained using a tissue culture has been reported sporadically in the literature, but SCoT-type markers were used for the first time in this study.

**Figure 3:**
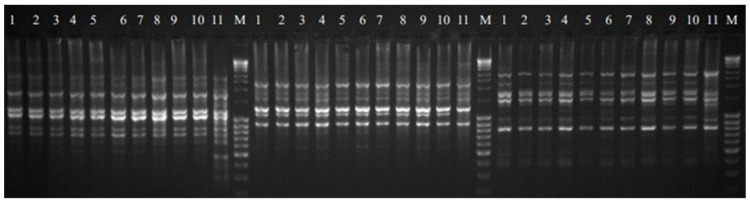
SCoT profile of regenerants 1–10 (from R-1to R-10 respectively) and DP—11 for primer 19, 24 and 25 respectively. M—NZYDNA Ladder III.

Eleven SCoT primers were analyzed in this study, and their sequences are listed in Table 3. The visualized amplification products after electrophoresis are shown in the images (Figure 3). All primers produced a total of 130 polymorphic bands, averaging more than 11 bands per primer. The percentage of polymorphism obtained ranged from 20 to 45%, averaging 34.86% for the eleven primers mentioned above. Previous studies in medicinal plants [35,36] used RAPD and ISSR markers and indicated the presence of somaclonal variation in callus tissue, as well as between the regenerants obtained from it [37]. Specific products generated by eight primers (SCoT 4, SCoT 14, SCoT 19, SCoT 24, SCoT 25, SCoT 35, SCoT 46 and SCoT 50) were also detected. A total of 11 products were obtained, with an average of 0.73 bands for all primers amplifying these products. All analyzed primers generated monomorphic products. Primer SCoT 46 produced the highest number of bands of this type, and SCoT 35 produced the lowest. The size of the obtained products ranged between 310 and 9800 bp.

**Figure 4:**
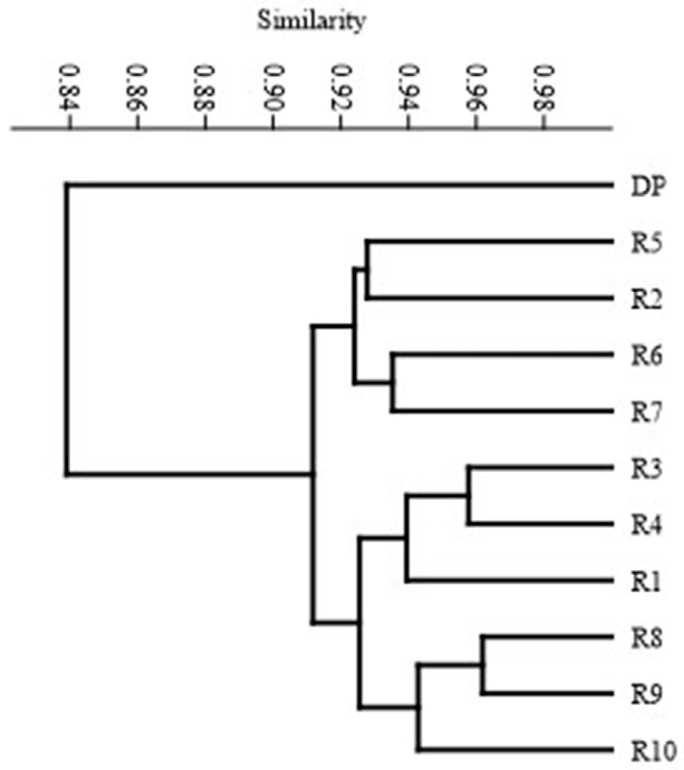
UPGMA dendrogram of DP and regenerants (from R-1 to R-10) based on SCoT marker analysis.

Based on the polymorphism identified using the SCoT technique, the genetic similarity between the studied genotypes was determined according to Dice’s formula after Nei and Li [12]. Table 4 shows the values of genetic similarity between the genotypes. The similarity of individual regenerants to the donor plant ranged from 0.82 to 0.86. Regenerates R3 and R8 showed the highest similarity to the donor plant (0.86), while R7 showed the lowest, at the level of 0.82. The highest similarity was found for regenerant R3 and R4, as well as R8 and R9, amounting to 0.96. A similarity below 0.9 was observed between regenerant R1 and R2; R2–R8; R2–R9. The mean similarity among the analyzed genotypes was 0.90, which indicated rather minor changes within the studied group of regenerants. The level of polymorphism in the range of 10–15% indicates low genetic variability for the SSR, RAPD and ISSR markers [35,36,38]. Etminan et al. [39] and Shahlaei et al. [40] reported that the verification of the results with SCoT markers could lead to different conclusions due to the higher informativeness of this marker compared to other marker systems. The variability observed in our study was due to the use of more sensitive markers, such as SCoT-type markers. Therefore, the utilization of this marker system in the identification of genetic variation in regenerants grown in in vitro cultures allows the detection of more subtle changes at the DNA level. The results are also visualized in the form of a dendrogram (Figure 4). It was observed that the studied genotypes could be assigned to two groups, which consisted of one single genotype (DP) and a cluster consisting of the remaining 10 regenerants. The presented method of grouping the studied genotypes clearly indicates the distinctiveness of DP in comparison to all in vitro regenerated plants. This confirms the existence of genetic variation within the analyzed genotypes, resulting from the process of indirect organogenesis used to obtain them. The reasons for this differentiation may result from in vitro culture conditions affecting different regions of the genome, including the types and level of PGRs use, which can stimulate rapid and disordered cell proliferation or the accumulation of somatic mutation during the tissue culture period [41,42,43].

## 4. Conclusions

In the literature, a number of works can be found indicating the possibility of regenerating shoots from nodal segments in various *Scutellaria* species, however, none of them analyzed the impact of regeneration through the callus stage (indirect organogenesis) on the variability within the obtained regenerants, and this has been presented in this study both at the phenotypic level and in detail at the genotypic level. The authenticated protocol presented in this work enables the micropropagation of *S. baicalensis*. The application of a protocol using indirect organogenesis generates somaclonal variation among the regenerants. The effective revealing of this variability at the DNA level was possible thanks to the precise SCoT marker system, which was presented for the first time for the studied species. This provides a basis for further biochemical studies of the obtained regenerants aimed at isolating genotypes with higher secondary metabolite content of *S. baicalensis*.

## Figures and Tables

**Table 1:** Phytohormones used in the media, their combinations and the observed effect on the regeneration process of *S. baicaliensis*.

Medium Number	Plant Growth Regulator Concentration and Combination (mg × dm^−3^)					Mean Number of Shoot per Explant	Mean Shoot Length (cm)	Mean Number of Nodes per Shoot	Calli Formation
	KIN	BAP	TDZ	GA_3_	2iP				
1.	0.5	0.5	-	-	-	7.4 a * (I)	1.7 b	3.0 b	+++
2.	-	-	0.5	-	-	-	-	-	+++
3.	-	-	-	0.5	-	2.1 b (D)	6.3 a	4.4 a	-
4.	-	-	-	-	0.5	6.0 ab (I)	5.5 a	3.5 ab	−/+
5.	-	-	-	0.5	0.5	4.8 ab (I)	4.8 a	3.2 ab	+

*—means in columns followed by the same letter do not differ significantly at 5% level of probability, D—direct organogenesis, I—indirect organogenesis, +++ very high degree of callus tissue formation, ++ high degree of callus tissue formation, + low degree of callus tissue formation, - no callus tissue formation; KIN-kinetin; BAP-6-Benzylaminopurine; TDZ-thidiazuron; GA_3_-gibberellic acid; 2iP-2-isopentenyladenine.

**Table 2:** Evaluation of the morphogenetic capacity of regenerants derived from medium number 1 regenerated on medium number 3.

Plant Number	Mean Number of Shoot	Mean Shoot Length	Mean Number of Nodes per Shoot	Root System Development Stage ^1^
R1	3.4 bc *	4.1 d	5.7 de	1
R2	4.6 b	6.0 c	8.4 c	3
R3	2.0 c	7.8 bc	7.0 cd	1
R4	3.4 bc	7.7 bc	6.7 cd	3
R5	2.0 c	13.3 a	16.5 a	2
R6	7.4 a	2.8 e	4.6 e	2
R7	9.4 a	3.2 de	4.0 e	3
R8	8.6 a	5.0 cd	5.4 de	2
R9	2.4 bc	8.7 b	10.5 b	3
R10	3.6 bc	8.4 b	6.9 cd	2
Mean	4.6	6.7	7.5	-
Coefficient of variation (%)	56.8	44.8	45.2	-

*—means in columns followed by the same letter do not differ significantly at 5% level of probability, ^1^—Root system development stage: 1—only a few short roots, 2—a few roots up to 2 cm long, 3—several roots longer than 2 cm.

**Table 3:** Assessment of the number and type of products generated with the primers used in the study.

Primer	Primer Sequence 5′-3′	Number of Products				% of Polymorphism	Size Range (bp)
		Total	Polymorphic	Specific	Monomorphic		
SCoT 4	CAACAATGGCTACCACCT	10	2	1	7	20.00	600–6000
SCoT 9	CAACAATGGCTACCAGCA	9	4	0	5	44.44	1200–5800
SCoT 14	ACGACATGGCGACCACGC	13	3	2	8	23.08	420–3200
SCoT 19	ACCATGGCTACCACCGGC	15	5	2	8	33.33	310–9300
SCoT 24	CACCATGGCTACCACCAT	11	5	1	5	45.45	520–9000
SCoT 25	ACCATGGCTACCACCGGG	10	3	1	6	30.00	370–6000
SCoT 31	CCATGGCTACCACCGCCT	12	5	0	7	41.67	470–8000
SCoT 35	CATGGCTACCACCGGCCC	8	3	1	4	37.50	380–9800
SCoT 36	GCAACAATGGCTACCACC	13	5	0	8	38.46	480–5500
SCoT 46	ACAATGGCTACCACTGAG	15	4	1	10	26.67	500–8200
SCoT 50	ACAATGGCTACCACTGGG	14	6	2	6	42.86	380–5000
Mean per primer		11.82	4.09	1.00	6.73	34.86	-
Total		130	45	11	74	-	310–9800

**Table 4:** Matrix of genetic similarity between the studied genotypes obtained on the basis of SCoT markers.

	R1	R2	R3	R4	R5	R6	R7	R8	R9	R10	DP
R1	1.00	0.86	0.94	0.94	0.92	0.90	0.90	0.92	0.92	0.92	0.85
R2		1.00	0.90	0.90	0.93	0.93	0.92	0.89	0.89	0.90	0.83
R3			1.00	0.96	0.93	0.91	0.94	0.95	0.93	0.93	0.86
R4				1.00	0.92	0.92	0.91	0.93	0.92	0.90	0.84
R5					1.00	0.92	0.92	0.92	0.92	0.92	0.83
R6						1.00	0.94	0.91	0.91	0.90	0.83
R7							1.00	0.95	0.93	0.93	0.82
R8								1.00	0.96	0.94	0.86
R9									1.00	0.95	0.84
R10										1.00	0.83
DP											1.00

## Data Availability

Not applicable.
